# Antibody Selection for Cancer Target Validation of FSH-Receptor in Immunohistochemical Settings

**DOI:** 10.3390/antib6040015

**Published:** 2017-10-18

**Authors:** Nina Möker, Solveig Peters, Robert Rauchenberger, Nicolae Ghinea, Christian Kunz

**Affiliations:** 1 MorphoSys AG, Discovery Alliance and Technologies, 82152 Planegg, Bavaria, Germany; moekern@gmail.com (N.M.); Solveig.Peters@morphosys.com (S.P.); robert.rauchenberger@online.de (R.R.); 2 Curie Institute, Inserm-Tumoral Angiogenesis Unit, Translational Research Department, Curie Hospital, 75005-Paris, France; nicolae.ghinea@curie.fr

**Keywords:** antibody validation, cancer marker validation, FSH, FSHR, immunocytochemistry, immunohistochemistry, kidney cancer, ovarian cancer, prostate cancer

## Abstract

Background: The follicle-stimulating hormone (FSH)-receptor (FSHR) has been reported to be an attractive target for antibody therapy in human cancer. However, divergent immunohistochemical (IHC) findings have been reported for FSHR expression in tumor tissues, which could be due to the specificity of the antibodies used. Methods: Three frequently used antibodies (sc-7798, sc-13935, and FSHR323) were validated for their suitability in an immunohistochemical study for FSHR expression in different tissues. As quality control, two potential therapeutic anti-hFSHR Ylanthia^®^ antibodies (Y010913, Y010916) were used. The specificity criteria for selection of antibodies were binding to native hFSHR of different sources, and no binding to non-related proteins. The ability of antibodies to stain the paraffin-embedded Flp-In Chinese hamster ovary (CHO)/FSHR cells was tested after application of different epitope retrieval methods. Results: From the five tested anti-hFSHR antibodies, only Y010913, Y010916, and FSHR323 showed specific binding to native, cell-presented hFSHR. Since Ylanthia^®^ antibodies were selected to specifically recognize native FSHR, as required for a potential therapeutic antibody candidate, FSHR323 was the only antibody to detect the receptor in IHC/histochemical settings on transfected cells, and at markedly lower, physiological concentrations (ex., in Sertoli cells of human testes). The pattern of FSH323 staining noticed for ovarian, prostatic, and renal adenocarcinomas indicated that FSHR was expressed mainly in the peripheral tumor blood vessels. Conclusion: Of all published IHC antibodies tested, only antibody FSHR323 proved suitable for target validation of hFSHR in an IHC setting for cancer. Our studies could not confirm the previously reported FSHR overexpression in ovarian and prostate cancer cells. Instead, specific overexpression in peripheral tumor blood vessels could be confirmed after thorough validation of the antibodies used.

## 1. Introduction

Follicle-stimulating hormone (FSH) is an important hormone responsible for growth, maturation and function of human reproductive system. In females, FSH in the ovaries is involved in folliculogenesis; it induces maturation of ovarian follicles and production of estrogen [1]. In males, FSH in the testes stimulates Sertoli cell proliferation, and supports spermatogenesis [2]. FSH is a glycoprotein heterodimer that binds and acts through the FSH-receptor (FSHR), a G-protein coupled receptor. FSHR possesses the distinctive pattern of seven transmembrane spanning domains. Its large extracellular domain (ECD) places it in a specific subgroup together with luteinizing hormone receptor (LHR) and thyroid stimulating hormone receptor (TSHR). This receptor domain is responsible for the high-affinity binding of hormones [3,4,5]. In adult humans, FSHR is expressed mainly in the granulosa cells of the ovary and the Sertoli cells of the testis [3]. It is minimally expressed by the endothelial cells of gonadal blood vessels [6,7]. Reverse transcriptase-polymerase chain reaction (RT-PCR) analyses have shown, unequivocally, that FSHR is transcribed in extra gonadal tissues as well, particularly in the human female reproductive tract and the placenta [8], in benign prostatic hyperplasia [9,10,11], prostate cancer [9,10,11], and ovarian cancer [12,13,14].

The focus of this study was to evaluate FSHR as a therapeutic target for ovarian, prostate, and renal cancer. A number of studies indicated FSHR as an attractive target, because of its lack of expression in healthy tissue (despite the above-mentioned expression in the reproductive tracts) and overexpression in malignant ovarian and prostate cancerous tissues [15,16,17,18]. However, divergent immunohistochemical findings have been reported for the FSHR protein expression and localization [10,12,15,19,20,21,22]. Unfortunately, in a number of the expression studies published, the immunohistochemistry (IHC) antibodies used were not indicated, or are no longer available.

Three different antibodies have been frequently used to study, immunohistochemically, the expression of FSHR in human tumors: sc-13935 [23,24,25,26,27], sc-7798 [27,28], and FSHR323 [15,20,21,22,29,30], and are claimed to be suitable tools for immunohistochemical analysis of cancer tissues. These antibodies were commercially obtained and validated for their suitability to detect FSHR expression in different tissues. Furthermore, since it is challenging to identify monoclonal antibodies that can be used as tools for validation of recombinant protein antigens or cell lines, we used anti-human FSHR Ylanthia^®^ antibodies raised against two different peptides of the hFSHR ECD (aa295-332). Material generated for specificity testing of the antibodies included: FSHR extracellular domain presented in virus-like particles (VLPs), as well as cells stably expressing hFSHR, which were validated for their surface expression of functional hFSHR. In addition, different cancer cell lines reported to endogenously express hFSHR have been tested. These included the ovarian cancer cell lines OVCAR-3 [31], and Caov-3 [32], as well as prostate cancer cell lines PC-3 [10,11,16] and DU145 [10].

## 2. Results

### 2.1. Generation of Cell Lines and VLPs for Antibody and FSHR Target Validation

**Figure 1:**
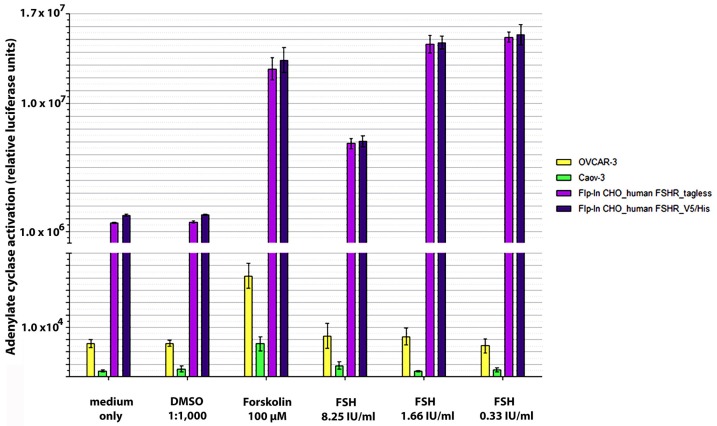
Detection of functional follicle-stimulating hormone (FSH)-receptor (FSHR) on the surface of different cell lines: Cyclic AMP signaling in response to FSH. Flp-In CHO cells expressing the hFSHR, OVCAR-3, and Caov-3 were treated with controls or different concentrations of FSH. The cAMP was measured by cAMP reporter gene assay (see Section 4). Controls: medium, dimethyl sulfoxide (DMSO) as negative controls; forskolin as assay positive control.

To ensure a reliable system for antibody validation, we aimed at setting up an antibody-independent measure for cell surface expression of functional FSHR protein. Biologically active FSHR ligand, human FSH, is commercially available because it is pharmaceutically produced for clinical fertility assistance. Cell surface expression of functional hFSHR was confirmed by induction of cyclic adenosine monophosphate (cAMP) signaling via human FSH in a cAMP-dependent reporter assay. As a positive control for the test system, we have used forskolin, which efficiently activates the cAMP reporter. In this set-up, all recombinant cell lines showed a strong dose-dependent response to human FSH (Figure 1).

Specificity of the assay was controlled by parental cell lines, which showed forskolin-induced reporter activity, but lacked a response to human FSH. Unexpectedly, none of the tested cancer cell lines showed a response to human FSH in the cAMP assay, although reporter activity could be activated by forskolin. Representative results obtained with ovarian cancer cell lines OVCAR-3 and Caov-3 are shown in Figure 1. Overall, these data indicate that only the generated recombinant cells, and not the tested cancer cell lines, expressed functional hFSHR protein on their surface.

In addition, virus-like particles (VLPs) were generated, as described in Material and Methods, which contain FSHR and the homologues receptors TSHR or LHR, in order to obtain a further layer of specificity analysis.

### 2.2. Validation of Anti-FSHR Antibodies

#### 2.2.1. Cell Binding Experiments

**Figure 2:**
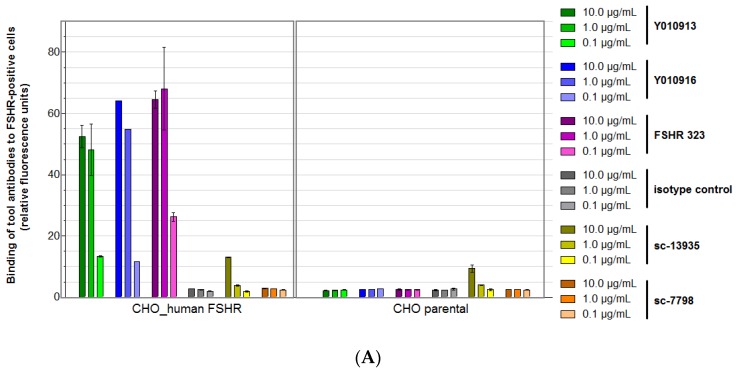

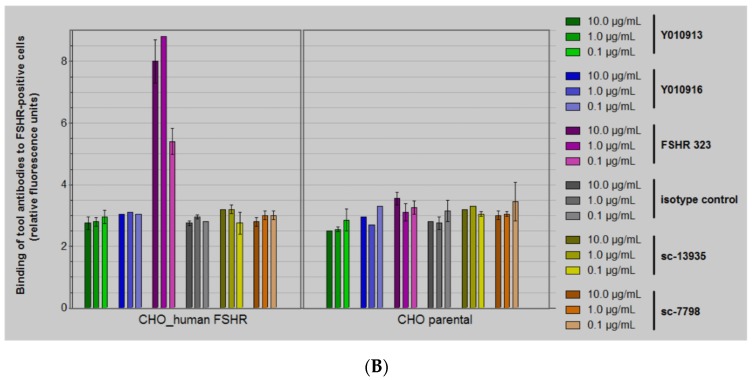
Binding of tool antibodies to cells expressing the recombinant human FSHR. Fluorescence-activated cell sorting (FACS) analysis using Flp-In CHO/hFSHR in comparison to the parental Flp-In CHO cells as specificity control. Different antibodies were tested for binding to cells overexpressing human FSHR versus parental cells not expressing human FSHR. Three antibody concentrations were tested (**A**); Antibody binding to cells was tested after fixation (formaldehyde treatment over-night) (**B**).

Antibodies used in this study are summarized in Table 1. As a first measure to analyze specificity to hFSHR, the Ylanthia^®^ antibodies Y010913 and Y010916, as well as the commercially available antibodies FSHR323 (INSERM), sc-7798, and sc-13935 (Santa Cruz), were characterized for binding to the previously validated cell lines by using flow cytometry. As shown in Figure 2A, both Ylanthia^®^ antibodies, as well as the tool antibody FSHR323, showed efficient and specific binding to CHO cells overexpressing hFSHR only.

In contrast, (i) no binding to cell-presented hFSHR was observed for sc-7798 in the flow cytometry analyses using unfixed (Figure 2A) or formaldehyde-fixed cells (Figure 2B); and (ii) strong unspecific binding of sc-13935 antibody to CHO cells not expressing hFSHR was noticed both before and after fixation with formaldehyde (Figure 2A,B, respectively). Therefore, the polyclonal antibodies sc-7798 and sc-13935 were ruled out as tool antibodies for human FSHR.

#### 2.2.2. Thorough Specificity Assessment

Three anti-FSHR antibodies previously used in expression analyses of cancerous tissues could be identified: sc-7798, sc-13935, and FSHR323. Santa Cruz antibodies sc-7798 and sc-13935 have already failed in our first lines of quality control (QC) analyses. For the remaining FSHR323 antibody, a thorough and in-depth characterization for specificity and suitability for immunohistochemistry was undertaken.

The antibodies Y010913, Y010916, and FSHR323 were subjected to protein panel profiling (3P), where unspecific binding to a panel of approximately 30 carefully selected, but non-target related proteins was assessed, as previously described by Frese and coworkers [33]. FSHR323, and the antibodies Y010913 and Y010916, passed the specificity quality control in this assay by binding to hFSHR ECD, but not to all of the other tested proteins (Figure S1A,B) One important measure for reliability of the antibody preparations is the protein quality, i.e., protein purity, aggregation, monomer content. This was assessed by reducing and non-reducing sodium dodecyl sulfate gel capillary electrophoresis (CE-SDS), as well as size exclusion chromatography. Our acceptance criteria for antibody quality were purity under reduced condition >95%, purity under non-reduced condition >80%, and monomer content in high-performance size-exclusion chromatography (HP-SEC) >90%. All three assessed antibodies passed these criteria (data not shown).

**Figure 3:**
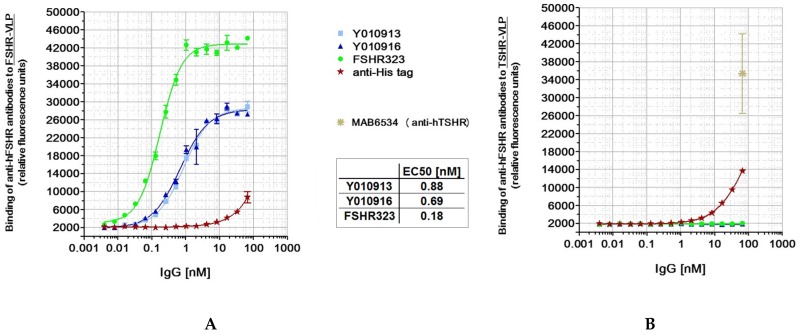
Specificity assessment of anti-FSHR antibodies using Virus-like particles containing FSHR in ELISA. VLPs containing the FSHR protein (**A**) or TSHR protein (**B**) used as control were coated on Maxisorp plates. FSHR was detected with different concentrations of Y010913, Y010916, and FSHR323. The mouse monoclonal anti-hTSHR antibody 6534 (R&D Systems) was used to detect VLPs containing hTSHR protein. The mouse monoclonal anti-His tag antibody was used as an irrelevant antibody.

The partly homologous glycoprotein hormone (GPH) receptors luteinizing hormone receptor (LHR) and thyroid stimulating hormone receptor (TSHR) were also published to be overexpressed in different cancers [34,35,36]. Therefore, it was critical to additionally ensure, that the selected antibodies did not bind to these other human GPH receptor proteins. Human FSHR, TSHR, and LHR were expressed as Virus-like particles (VLPs), and used in ELISA studies with the antibodies to be validated. As illustrated in Figure 3A, the Ylanthia^®^ antibodies Y010913 and Y010916, as well as FSHR323, bound in a dose-dependent manner to hFSHR (EC_50_ (nM): 0.8796, 0.6923, and 0.1754, respectively), and did not cross-react with any of the two other glycoprotein hormone receptors, hTSHR (Figure 3B) and hLHR.

#### 2.2.3. Competition of Antibodies with FSH for Binding to the FSHR

**Figure 4:**
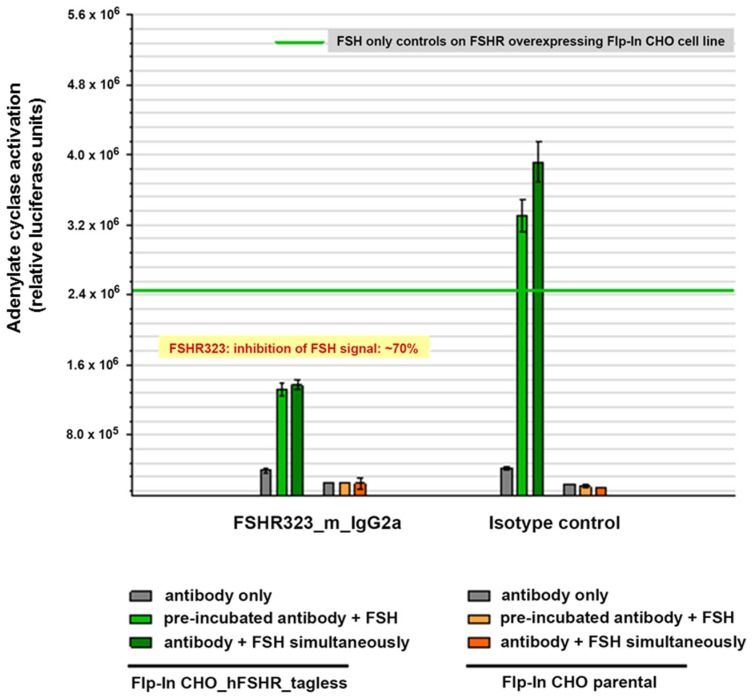
Cyclic AMP signaling in response to FSH after pre-incubation with isotype control IgG and FSHR323 antibody. Flp-In CHO/FSHR cells expressing the recombinant hFSHR were plated and incubated with various concentrations of monoclonal antibodies in DMEM and 0.5 mM 3-Isobutyl-1-methylxanthin (IBMX). FSH (1.65 nM) was added. Cyclic AMP was measured by cAMP reporter gene assay (see Section 4). FSHR323 inhibited cAMP-dependent signaling in response to FSH.

In order to further clarify the specificity of the FSHR323, we tested the antibody in an in vitro functional assay. We compared the effect of pre-incubation of FSHR-expressing cells with the antibody on FSH-induced signaling in the cAMP assay. It turned out that FSHR323 reduced the FSH-induced signaling by ~70%, indicating a competition with FSH for the binding to the receptor (Figure 4). This result was in agreement with the ability of FSHR323 to inhibit FSH activation of the receptor [6].

#### 2.2.4. IHC Pre-Experiments Analyzing Antibody Binding to Cells after Fixation 

A potential prediction for IHC suitability of antibodies is the comparison of binding to cell-presented receptor protein before and after fixation conditions best corresponding to those applied for the tissues of interest. In this case, cells were either used without (*unfixed*) or with overnight treatment with 4% formaldehyde (*fixed*). Fixation of the cells led to masking of the binding epitope for Y010913 and Y010916. In contrast, the binding epitope of FSHR323 was sufficiently retained for efficient antibody binding (Figure 2B).

**Figure 5:**
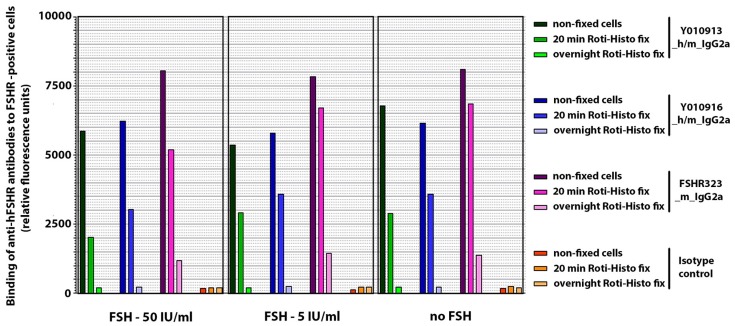
Effect of FSH on binding of FSHR323, Y010913, and Y010916 to fixed cells expressing FSHR. FSH was added in excess to the FSHR expressing cells, followed by fixation with PBS-buffered paraformaldehyde for 20 min or overnight. Non-fixed cells were used as a positive control. A non-related mouse IgG2a antibody served as negative control.

It is well documented that the FSH levels are elevated in ovarian cancer [37,38]. Because the binding of FSHR323 to cancer tissue may be impaired by the presence of FSH during tissue fixation, it was necessary to perform a pre-experiment addressing this scenario. FSH was added in excess to the FSHR expressing cells, followed by a fixation with PBS-buffered paraformaldehyde for 20 min or overnight. Non-fixed cells were used as a positive control. A non-related mouse IgG2a antibody served as negative control. As shown in Figure 5, the presence of FSH did not result in reduced binding of FSHR323, or Y010913 and Y010916, respectively to non-fixed cells.

However, the cell binding of all three antibodies was reduced after fixation, and the reduction was fixation time-dependent. Indeed, FSHR323 showed reduced binding capacity after overnight fixation of the cells (17% remaining), although not to the extent of Y010913 and Y010916 (3.6% and 3% remaining, respectively). This indicated that the epitopes of FSHR323, Y010913, and Y010916 were masked in the process of fixation.

**Figure 6:**
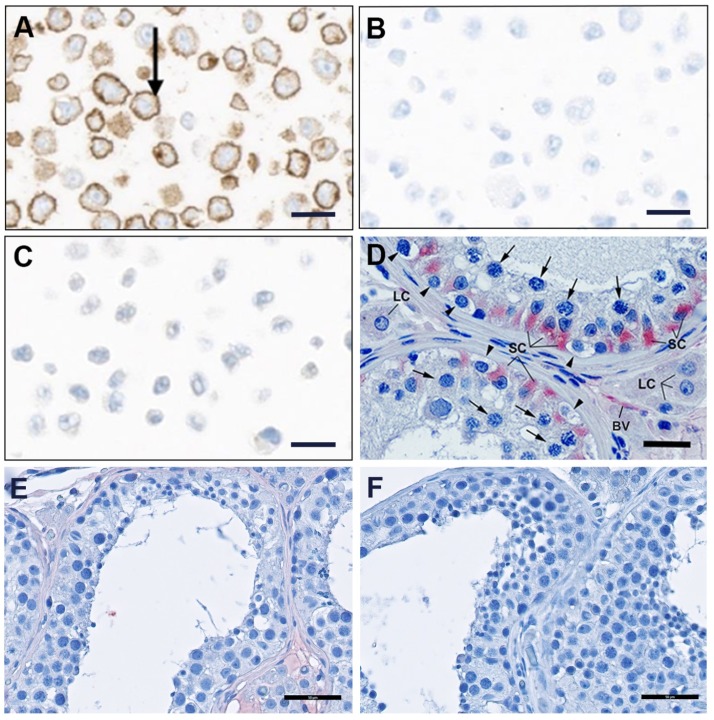
Immunocytochemical and immunohistochemical detection of FSHR on paraffin embedded cells and tissues. (**A**–**C**): Binding of FSHR323 to cell-presented hFSHR. Immunocytochemical analysis was performed on paraffin-embedded sections of Flp-In CHO/FSHR using FSHR323 antibody, followed by a secondary peroxidase-coupled antibody visualized by the brown peroxidase-reaction product of diaminobenzidine (DAB). (**A**): Strong membranous anti-FSHR staining of Flp-In CHO/FSHR (arrow); (**B**): The isotype control (mouse IgG2a) was completely negative; (**C**): No staining was seen in parental Flp-In CHO. D-F: FSHR expression in human testis tissue; (**D**): The receptor was detected by immunohistochemistry using the mouse FSHR323 monoclonal antibody followed by a secondary peroxidase-coupled antibody visualized by the red peroxidase-reaction product of aminoethyl carbazole (AEC). This method indicated that, in seminiferous tubules, only Sertoli cells (SC) express FSHR. Testicular blood vessels (BV) were also positive. Spermatogonia (arrowheads), pachytene spermatocytes (arrows), and Leydig cells (LC) were FSHR-negative. No FSHR-signal was visible in human testis with sc-7798 (**E**) and sc-13935 (**F**) antibodies. The scale bar represents 20 µm in panels A, B, and C, 25 µm in panel D, and 50 µm in panels E and F.

After application of different epitope retrieval methods (i.e., heat induced epitope retrieval in 10 mM citrate buffer, pH = 6 or 10 mM Tris, pH = 9, containing 1 mM EDTA, or protease treatment), the ability of antibodies to stain the paraffin-embedded Flp-In CHO/FSHR cells was tested. The monoclonal chimeric (human/mouse) anti-FSHR antibody clones Y010913 and Y010916 exhibited no reactivity with FSHR in the stable transfected Flp-In CHO/FSHR cell line (data not shown). In contrast, the FSHR323 antibody showed specific staining of membranes of Flp-In CHO/FSHR cells embedded in paraffin blocks (Figure 6A). The isotype control (mouse IgG2a) antibody was completely negative (Figure 6B). Also, no staining was seen in parental Flp-In CHO cells (Figure 6C).

#### 2.2.5. Immunohistochemical Detection of FSHR in Human Testis

Since the Flp-In CHO/FSHR cells may express non-physiologically high levels of receptor protein, it was necessary to verify that FSHR323 antibody was also able to detect hFSHR under physiological conditions. As illustrated in Figure 6D, all Sertoli cells (the FSH-target cells) of the human seminiferous epithelium were strongly immunostained using FSHR323. Spermatogonia, pachytene spermatocytes, and Leydig cells were FSHR-negative. Endothelial cells of testicular blood microvessels were also stained. This staining was not observed when FSHR323 antibody was replaced by the Santa Cruz antibodies sc-7798 (Figure 6E) and sc-13935 (Figure 6F).

Taking all quality controls into account, the antibody FSHR323 turned out to be highly specific and suitable for validation of hFSHR as a potential cancer target by immunohistochemistry. Therefore, this antibody was used to validate the FSHR expression in ovarian cancer, prostate cancer, and renal cancer tissues.

### 2.3. Characterization of FSHR as a Target for Human Cancer

**Figure 7:**
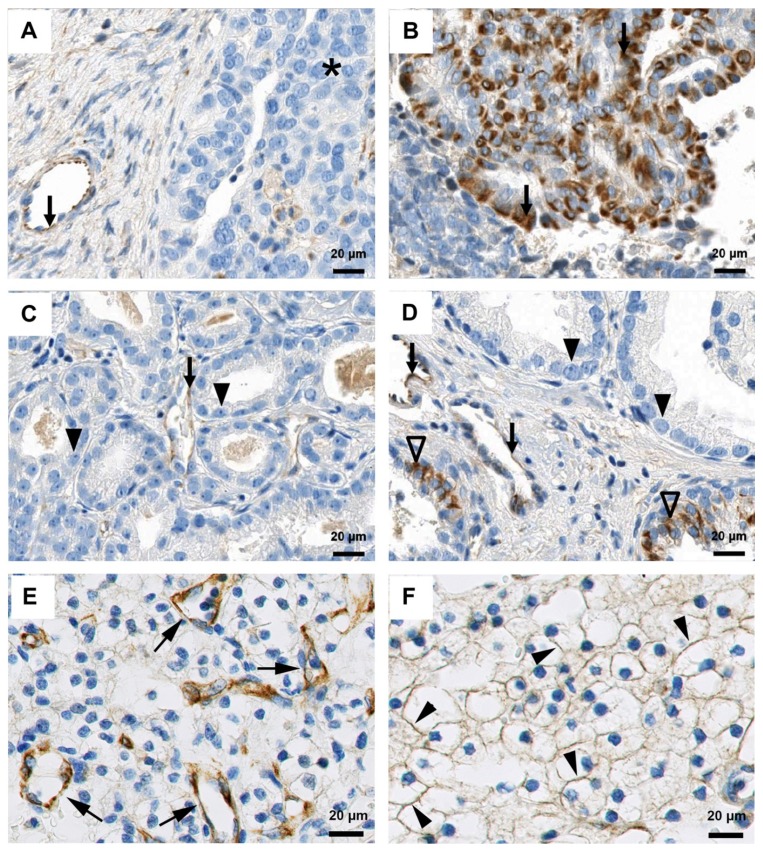
FSHR expression in human cancer detected with FSHR323 antibody. (**A**) and (**B**): Human ovarian cancer tissue. (**A**): No anti-FSHR staining of tumor cells (asterisk) was observed in 78% of patients. A weak to moderate cytoplasmic staining of small blood vessels was detected (arrows). (**B**): Sample exhibiting a strong cytoplasmic anti-FSHR staining of tumor cells (arrows). (**C**) and (**D**): Human prostate cancer tissue of Gleason grade of 3. (**C**): Sample exhibiting no anti-FSHR staining in tumor cells (arrowheads), but a weak cytoplasmic staining of small vessels in the tumor stroma (arrows). (**D**): No anti-FSHR staining of tumor cells was observed (arrowheads). A weak to moderate cytoplasmic staining of small vessels in the tumor and the peripheral stroma was noticed (arrows). Some of the tumor-adjacent hyperplastic prostate gland epithelial cells showed a strong cytoplasmic anti-FSHR staining (open arrowheads). (**E**) and (**F**): Human kidney cancer tissue. Arrows point to FSHR-positive blood vessels. Arrowheads indicate a moderate membrane staining of renal cancer cells.

All 32 samples of ovarian cancer showed positive staining of small vessels with staining intensities ranging from weak to strong. Interestingly, compared to tumor stroma, the FSHR signal was stronger in vessels of peripheral stroma (Figure 7A). If present in the analyzed sample, normal ovarian cortical stroma was FSHR-negative. Staining of tumor cells was only observed in 7 out of 32 samples (22%) (2 of weak intensity score (IS), 2 moderate IS, and 3 strong IS) (Figure 7B). The observed staining pattern was mainly heterogeneous. In terms of subcellular localization, the observed FSHR staining was pre-dominantly cytoplasmic, whereas the plasma membrane was only stained in single tumor cells.

The evaluation of 30 prostate cancer samples showed positive staining of small vessels. No difference between small vessels in the tumor stroma and the stroma of the tumor-adjacent normal tissue was observed. None of the 30 prostate cancer samples showed FSHR staining of the tumor cells. However, in four prostate cancer samples, the epithelium of tumor-adjacent normal or hyperplastic prostate glands (all samples included areas of adjacent normal prostate tissue) focally exhibited a strong cytoplasmic anti-FSHR staining. Representative pictures of these FSHR staining patterns for prostate cancer are illustrated in Figure 7C,D. In the case of kidney cancer, 29/31 samples (93.5%) showed positive staining for FSHR in small vessels (Figure 7E), and in 3/31 (10%) samples, a positive FSHR signal was detected in cancer cells (1 weak IS, 1 moderate IS (Figure 7F), and 1 strong IS). The tumor core vessels were negative or weakly positive. A moderate to strong FSHR323 staining was detected on blood vessels associated with the peritumoral benign tissue. The isotype controls were mainly negative, but in a few cases, non-specific staining of single tumor cells, macrophages or lymphoid cells was observed.

These results were in agreement with the ability of FSHR323 to detect specifically FSHR in cancer tissues as previously described [15,20,21,22,29,30]. However, overexpression of FSHR protein in tumor cells of ovarian or prostate cancer tissues, as previously described in several studies [10,11,12,19], could not be confirmed here.

## 3. Discussion

Since divergent immunohistochemical findings have been reported for the FSHR protein expression and localization in ovarian cancer and prostate cancer [10,11,12,13,14,15,16,17,18,19], our goal was to validate FSHR as a drug target in human cancers. The target validation for FSHR proved challenging for three reasons: (i) numerous publications about cancer cell lines or tissues expressing hFSHR did not specify the exact antibody used for analyses; (ii) a number of purchased antibodies could either only be used for certain applications (e.g., ELISA), did not recognize cell-presented hFSHR, or were unspecific [26]; and (iii) the antibodies used in two studies [10,11] for IHC detection of FSHR in the tumor cells of prostate adenocarcinomas are no longer available. We therefore decided to produce our own tools for the validation of antibodies that have been used in previous publications for FSHR immunohistochemistry with cancerous tissues. To avoid false-positive results due to non-specific binding to tissue/cell components or recognition of epitopes shared by several molecules [39], these antibodies needed to be tested in various control experiments. The specificity criteria for selection of antibodies were (i) binding to hFSHR overexpressing CHO-cell lines; (ii) recombinant hFSHR purified in virus-like particles, hFSHR ECD (purified from *Escherichia coli*), no binding to parental cells and a set of recombinant proteins in protein panel profiling (3P); and (iv) quality control of the protein preparation (protein purity, aggregation, monomer content). As quality control for specific antibody binding, we used two potential therapeutic anti-hFSHR Ylanthia^®^ antibodies (Y010913, Y010916). From the five tested anti-FSHR antibodies, only Y010913, Y010916, and FSHR323 showed specific binding to cell-presented hFSHR.

The ability of Y010913, Y010916, and FSHR323 antibodies to stain the paraffin-embedded Flp-In CHO/FSHR cells and human cancer tissues was tested at Indivumed, a German integrated oncology company leveraging its leading oncology biorepository expertise with its pre-clinical, clinical, and diagnostic laboratory expertise. FSHR323 was the only antibody able to stain hFSHR immunocytochemically in transfected cells, and to detect it immunohistochemically at the markedly lower physiological concentrations (e.g., FSHR in Sertoli cells of human testes).

Concerning the validation of tumor-related FSHR expression, the present data indicated that only a minority of tissue samples derived from patients suffering from ovarian, prostate or kidney cancer showed FSHR-positive staining in tumor cells. If tumors were positive, in most cases, only a minor portion of the cancer cells showed FSHR expression, mainly in the cytoplasm and rarely on the plasma membrane. Thus, the favorable tumor cell expression profile of FSHR previously described for ovarian cancer [10,11,12,19] could not be confirmed. In contrast, the expression of FSHR on blood vessels at the periphery of different tumors [15,20,21,30] could be confirmed. The endothelial FSHR expression on blood vessels of tumor stroma and peripheral tumor stroma might be involved in vascular remodeling at tumor periphery [21,22], but the role of FSHR in this process is not clear.

The need for reliable antibodies, especially in the biomedical research, has been recently emphasized by several researchers (summarized in [40,41]). This study serves as another example for the confusion that data generation with antibodies that were either not named in publications, or not sufficiently target-specific, can cause.

This study serves as another example of the confusion that can be caused by generation of biomedical data with the use of antibodies that are either not listed by name and clone identifier in publications, or not sufficiently assessed for target specificity and reproducibility.

The present study highlights, again, how insufficiently characterized and/or non-target specific antibodies can cause confusion during data generation.

## 4. Material and Methods

### 4.1. Human Tissue Specimens

Paraffin sections for ovarian cancer (32 patients) and prostate cancer (30 patients) were obtained from the Biorepository of Indivumed, Hamburg, Germany. Thirty-one kidney cancer tissues were from the Biorepository of Asterand, Royston, Hertfordshire, UK. Paraffin sections for normal human testis (3 donors) were from the Biorepository of Curie Hospital, Paris.

### 4.2. Cell Lines and Cell Culture

Human cancer cell lines used in this study were ovarian cancer cell lines OVCAR-3 (ATCC^®^ HTB-161™, LGC Standards GmbH, 46485 Wesel, Germany) and Caov-3 (ATCC^®^ HTB-75™), as well as prostate cancer cell lines DU 145 (ATCC^®^ HTB-81™) and PC-3 (ATCC^®^ CRL-1435™, LGC Standards GmbH, 46485 Wesel, Germany). Mammalian host cell line used for receptor cell surface expression was Flp-In™-CHO (Invitrogen # R75807, Thermo Fisher Scientific Inc., Waltham, MA USA 02451) stably expressing the *lacZ-Zeocin™* fusion gene from pFRT/*lac*Zeo2. OVCAR-3 cells were cultured and maintained in Roswell Park Memorial Institute medium (RPMI-1640, Gibco #21875-091, Thermo Fisher Scientific Inc., Waltham, MA, USA 02451) supplemented with 10% fetal bovine serum (Sigma #F7524-500 ML; heat inactivated 56 °C, 30 min). Caov-3 and DU145 cells were cultured and maintained in Dulbecco’s Modified Eagle Medium (DMEM, Gibco #10938-025) supplemented with 10% fetal bovine serum (Sigma #F7524-500 ML; heat inactivated 56 °C, 30 min), 1× GlutaMAX (Gibco #35050-087) and 1× sodium pyruvate (Gibco #11360-039). PC-3 cells were cultured and maintained in Ham’s F-12K (Kaighn’s) Medium (Gibco #21127-022, Thermo Fisher Scientific Inc., Waltham, MA USA 02451) supplemented with 10% fetal bovine serum (Sigma #F7524-500 ML; heat inactivated 56 °C, 30 min). Flp-In™-CHO cells were cultured and maintained in Ham’s F12 Nutrient Mixture Medium (Gibco #21765-037) supplemented with 10% fetal bovine serum (Sigma #F7524-500 ML; heat inactivated 56 °C, 30 min) and 100 µg/mL Zeocin (Invivogen #ant-zn-5p). Flp-In™-CHO cells stably expressing the receptor of interest were cultured and maintained in Ham’s F12 Nutrient Mixture Medium (Gibco #21765-037) supplemented with 10% fetal bovine serum (Sigma #F7524-500 ML; heat inactivated 56 °C, 30 min) and 600 µg/mL Hygromycin B (Invitrogen #10687-010). All cells were incubated in a humidified incubator at 37 °C in the presence of 5% CO_2_.

### 4.3. Construction of Expression Vectors Encoding hFSHR

The cDNA encoding hFSHR (Gene ID: 2492) was synthesized in two different versions. (1) A tagless variant comprising of a Kozak consensus sequence, adenine thymine guanine (ATG) initiation codon, and the natural signal peptide leader sequence upstream of the receptor encoding protein (including a stop codon), flanked by restriction enzymes recognition sites for cloning into expression vector. (2) As additional control for membrane localization of recombinantly expressed FSHR, a tagged variant containing a Kozak consensus sequence, ATG initiation codon, and the vkappa leader sequence followed by a V5 tag, tag linker, and a His6 tag upstream the FSHR sequence (including a stop codon), also flanked by restriction enzymes recognition sites for cloning into expression vector was constructed. Those complementary DNA (cDNA) inserts were subcloned into the pcDNA™5/FRT/TO expression vector (Invitrogen # V6520-20, Thermo Fisher Scientific Inc., Waltham, MA, USA 02451) via HindIII and XhoI restriction enzymes. Experiments for cell line characterization and specificity assessment of antibodies were performed with both resulting cell lines.

### 4.4. Generation of Potentially Therapeutic Antibodies against Human Follicle Stimulating Hormone Receptor 

To identify a highly specific anti-hFSHR antibody, we isolated hFSHR-targeted Fabs using MorphoSys’ Ylanthia^®^ phage-display technology [42] against a number of synthetic, biotinylated hFSHR peptides. In order to avoid competitive binding with FSH, the peptide sequences were designed to represent an area outside the binding domain for FSH. Different peptides representing the stretch of residues 295–330 [43] were used as antigens in three panning rounds. Enriched Fabs were screened for specificity to the hFSHR peptides. From the confirmed hits, a subset of clones was cloned into human IgG1 and chimeric human/mouse IgG2a format, and analyzed for binding to hFSHR expressed on different cell types.

### 4.5. Generation of Cells Stably Expressing FSHR

Stable Flp-In™ CHO expression cell lines were generated by co-transfection of Flp-In™ expression construct (pcDNA™5/FRT/TO_FSHR) and the pOG44 plasmid (Invitrogen #V6005-20). Co-transfection was done using lipid-mediated transfection (Lipofectamine; Invitrogen #11668, Thermo Fisher Scientific Inc., Waltham, MA, USA 02451) according to supplier′s manual. Stable transfectants were selected using 600 µg/mL hygromycin B (Invitrogen #10668). Cell surface expression of follicle stimulating hormone receptor (FSHR) was validated using flow cytometry or cAMP assays (see below).

### 4.6. Generation of VLPs Containing Full-Length hFSHR, hTSHR, or hLHR

Viral-like particles (VLPs) of human FSHR, TSHR, or LHR were generated by expression of human FSHR (aa16-695), human TSHR (aa21-764), or human LHR (aa27-699) coupled to GAG (HV1B1) in HEK293 cells. Transfection of the respective target protein encoding plasmid DNA was done using lipid-mediated transfection (Lipofectamine; Invitrogen #11668). Duration of the transient expression was three days. Cell culture supernatants were pre-cleared by centrifugation, and afterwards, filtered through a 0.45 µm filter unit (Corning^®^ 1000 mL Vacuum Filter/Storage Bottle System, #430516, Corning Inc., Corning, NY, USA 14831). The VLP containing supernatant was purified by Immobilized metal ion affinity chromatography (IMAC) affinity chromatography on an ÄKTA Avant 150 (GE Healthcare) HPLC system with a self-packed POROS^®^ MC column (Applied Biosystems #1542802, Thermo Fisher Scientific Inc., Waltham, MA, USA 02451). After elution, the buffer was finally exchanged to 1× PBS and size and purity of the preparations was checked by sodium dodecyl sulfate polyacrylamide gel electrophoresis (SDS-PAGE) and DLS (diffractive-light scattering).

### 4.7. Antibodies

Anti-hFSHR antibodies used in this study are summarized in Table 1.

### 4.8. ELISA Assays

For detection of anti-hFSHR antibody binding, biotinylated peptides used for pannings were coated on 384-well Neutravidin plates (Thermo Scientific #15402) (1 µg/mL in 1× PBS (Gibco #14190) at 4 °C overnight, and subsequently blocked for two hours at room temperature with 5% bovine serum albumin (BSA) (Sigma #A-7906) in 1× PBS. For binding studies of antibodies to VLPs containing hFSHR, hTSHR, or hLHR, 384-well Maxisorp™ plates (Nunc #460518, Thermo Fisher Scientific Inc., Waltham, MA, USA 02451) were coated with the appropriate VLP preparation at 4 °C overnight, and subsequently blocked for two hours at room temperature with 5% milk powder (Saliter) in 1× PBS. Test antibodies were incubated for one hour at room temperature. Plates were washed and antibody binding was detected using alkaline phosphatase linked goat anti-mouse (Jackson-Immuno-Research #115-055-003) or goat anti-rabbit (Sigma #A3687) secondary antibodies followed by AttoPhos^TM^ fluorescence substrate (Roche #1484281).

### 4.9. Flow Cytometry

The binding of antibodies to hFSHR expressed on mammalian cells was assessed by flow cytometry. Cells were cultured for 3 days, harvested with Versene (Gibco #15040) and diluted to 1 × 10^6^ cells/mL in Superblock Blocking Buffer (ThermoScientific #37515). 1 × 10^5^ cells were added to wells of a 96U- well plate (Nunc #163320) and blocked on ice for one hour. After centrifugation at 250× *g* for 3 min at 4 °C, supernatant was removed and primary antibodies were added to the pelleted cells and incubated for 1 h on ice. The cells were washed and pelleted two times with 100 µL FACS buffer consisting of 3% fetal bovine serum (FBS)/0.02% sodium azide in 1× PBS (Gibco# 14190). Mouse_IgG2a and human/mouse_IgG2a were detected using phycoerythrin (PE) conjugated goat anti-mouse IgG (Jackson Immuno Research #115-116-071). After incubation of 30 min on ice, two additional washing steps were performed, followed by a final centrifugation step at 250× *g* for 3 min at 4 °C. Finally, cells were resuspended in 150 µL FACS buffer and fluorescence values were measured with a FACS Array (BD Biosciences). Cell surface bound anti-hFSHR antibody was assessed by measuring the median intensity fluorescence.

### 4.10. Fixation

For fixation, cells were incubated with 4% formaldehyde (Roti^®^-Histofix, Roth #P087.5, Carl Roth GmbH + Co. KG, 76185 Karlsruhe, Germany) overnight at room temperature, prior to flow cytometry as described above.

#### Fixation in Presence of FSH

In IHC studies, FSHR expressing cells might be loaded with FSH. The ability of binding of anti-FSHR antibodies to FSH pre-loaded cells was again assessed by flow cytometry. FSHR expressing Flp-In CHO cells were harvested as described above (chapter “flow cytometry”) followed by a pre-incubation step with FSH (Bravelle 75 IU, Ferring Arzneimittel, PZN-4482639) for one hour on ice prior to a potential fixation (chapter “Effect of paraformaldehyde fixation on antibody binding to hFSHR-positive cells”) of cells. Analysis of cell binding capacity was done as described above (chapter “flow cytometry”).

### 4.11. FSH-Mediated Stimulation of Adenylate Cyclase

Mammalian cell lines used for adenylate cyclase reporter gene assay were Flp-In™-CHO cells stably overexpressing hFSHR in tagged and tagless variants. Cell lines were transfected with pGL4.29(luc2P/CRE/Hygro) Vector (Promega #9PIE847) containing a cyclic adenosine monophosphate (cAMP) response element (CRE) that drives the transcription of the luciferase reporter gene luc2P. Twenty-four hours prior to transfection, 2 × 10^4^ cells per well (in antibiotic-free growth medium) were seeded into a 96-well flat bottom assay plate (Costar #3903). Transfection was done using lipid-mediated transfection (*Trans*IT-LT1, Mirus #MIR2304) according to supplier’s manual. Twenty-four hours after transfection, cells were stimulated with 0.33 IU/ml of FSH (Bravelle 75 IU, Ferring Arzneimittel, PZN-4482639) and luciferase activity was measured using Bright-Glo Luciferase Assay System (Promega #E2620). During the five hours of FSH-mediated cAMP induction, cells were incubated in the presence of 5% CO_2_ in a humidified incubator at 37 °C.

To test if anti-hFSHR antibodies have any effect on FSH-mediated stimulation of adenylate cyclase, antibodies were pre-incubated with cells for one hour or added directly with FSH to the pGL4.29 (luc2P/CRE/Hygro) transfected cells. Readout was performed as described above.

### 4.12. Immunocytochemistry and Immunohistochemistry

Immunocytochemistry and immunohistochemistry studies were performed at Indivumed, Hamburg, Germany. Briefly, the formaldehyde fixed, paraffin embedded (FFPE) samples were sliced into 3 μm sections and mounted on positively charged SuperFrost Ultra Plus glass slides (Roth). IHC was implemented on the Discovery XT staining platform (Roche Diagnostics/Ventana Medical Systems). FFPE slides were deparaffinized within the staining instrument and immunostained using the ChromoMap DAB/AEC Detection Kit (Roche Diagnostics). The detailed staining procedure is described in Table 2.

Afterwards, slides were manually washed using hot water supplemented with detergent, followed by tap water only and dH_2_O in a final step. For dehydration, the slides were transferred to an ascending ethanol series (2 times 80%, 2 times 96%, 2 times abs. EtOH; 3 min each). After dehydration, the slides were transferred to xylene (3 times 2 min) and automatically embedded in Pertex.

### 4.13. Histopathological Evaluation 

Tumor contents were estimated using H&E-stained sections. For the evaluation of anti-hFSHR staining of tumor cells, an intensity score (IS) specifying negative (0), weak (1+), moderate (2+), or strong (3+) staining was used. The evaluation further included the specification of the percentage of positively stained tumor cells (PC), the predominant staining intensity, as well as the staining pattern. Within the tumor area, a distinction was made between a homogeneous and a heterogeneous staining pattern. A homogeneous staining pattern thereby implied a constant staining intensity (weak, moderate, or strong) of tumor cells, whereas a heterogeneous staining pattern implied varying staining intensities of tumor cells within the same tumor area. Furthermore, the predominantly stained subcellular compartment (m: membrane, n: nucleus, c: cytoplasm) of tumor cells was determined. In addition to the evaluation of anti-FSHR stained tumor cells, the intensity (weak, moderate or strong) of anti-FSHR stained tumor vessels was determined. To evaluate non-specific staining, an intensity score that specified negative (0), weak (1+), moderate (2+), or strong (3+) staining was applied to the isotype controls. In case of positive staining, the stained cell type was determined.

### 4.14. Specificity Analysis Using Protein Panel Profiling Assay

Analysis of overall specificity of IgG molecules was basically performed as described by Frese et al. [35]. However, the panel of the coated proteins had been slightly adapted. Recombinant FSHR from MyBioSource (#MBS965616) was used as a positive binding control.

Briefly, binding of IgG molecules at concentrations of 10 nM and 100 nM to coated proteins in Meso Scale Discovery (MSD) 384-well standard microtiter plates (#L21XA) was analyzed by electrochemiluminescence (ECL)-based readout. ECL binding signals of sample IgG molecules (mouse IgG2a, human/mouse IgG2a and human IgG1f formats) were compared to binding signals of a well-characterized reference antibody (MOR3207 anti-lysozyme human IgG2a and human/mouse IgG2a formats, respectively), and binding ratios were calculated. Overall specificity of sample IgG molecules was assessed by total number of binding hits to coated non-target-related proteins, and sum of cumulative binding ratios. For further details, refer to Frese et al.

## 5. Conclusions

Taking all quality controls into account, our results indicated that only antibody FSHR323 proved suitable for target validation of FSHR in an immunohistochemical setting for cancer, inciting us to think that only the IHC results from the previous studies using this particular antibody are to be trusted. Indeed, our results suggest that, in accordance to previously published data [15], FSHR is not a suitable target for monoclonal antibody therapy with direct targeting of ovarian, prostate, or renal cancer cells. Instead, our data confirm the localization of FSHR in endothelial cells at the periphery of ovarian, prostate, and renal cancer. At this location, blood vessels are essential for tumor growth and metastasis, because they make connections between the normal circulatory system of the patient and tumor core vessels [21]. Therefore, occlusion/collapse of FSHR-positive peritumoral connecting vessels may halt blood flow towards tumors, resulting in the death of tumor cells from lack of oxygen and nutriments. Since FSHR is a common marker of peritumoral vessels [15,30], a single therapeutic agent may, in principle, be applicable to a wide range of tumor entities. Combining FSHR-target therapy with anti-proliferative anti-tumor therapies and/or angiogenesis inhibitors may lead to additive or synergistic activity in malignant tumors.

## Figures and Tables

**Table 1:** Anti-hFSHR antibodies used in this study for cancer target validation of FSHR.

Antibody	Specificity	Description	Isotype	Application (Acc. to Supplier)	Antigen	Supplier
sc-7798	anti-hFSHR	Goat polyclonal		WB, ELISA,IF, IP	Peptide map-ping near the *N*-terminus of hFSHR	Santa Cruz
sc-13935	anti-hFSHR	Rabbit polyclonal		WB, ELISA,IF, IP	aa1-190	Santa Cruz
FSHR323	anti-hFSHR	Mouse monoclonal	IgG2a	WB, ELISA,IF, IP, ICC, IHC		Obtained from INSERM
Y010913	anti-hFSHR	Human/mouse monoclonal	IgG2a			Ylanthia^®^ antibody
Y010916	anti-hFSHR	Human/mouse monoclonal	IgG2a			Ylanthia^®^ antibody
Isotype control	Irrelevant antigen	Human/mouse monoclonal	IgG2a		irrelevant non-human protein	HuCAL^®^ antibody

WB: Western Blot, IF: Immunofluorescence, IP: Immunoprecipitation.

**Table 2:** Anti-FSHR staining conditions by using automated IHC research slide staining system.

Staining Instrument	Discovery XT
Fixation	4% Paraformaldehyde
Epitope retrieval	CC1 Mild (Cell conditioning solution 1, EDTA buffer); 30 min, 100 °C
Blocking	Normal goat serum diluted (Dispenser OPTION 4), 1:50, 8 min
Dilution buffer	DCS antibody diluent
Primary antibody	FSHR323, 2 µg/mL, 1 h, 37 °C
Secondary antibody	OmniMap DAB /AEC Kit
Counterstain	Hematoxylin II, 8 min; Bluing Reagent, 4 min

## Supplementary Materials

The following are available online at http://www.mdpi.com/2073-4468/6/4/15/s1, Figure S1: Analysis of overall specificity of IgG molecules using Protein Panel Profiling (3P).
