# Correction: Moeker, N.; et al. Antibody Selection for Cancer Target Validation of FSH-Receptor in Immunohistochemical Settings. *Antibodies* 2017, *6*, 15

**DOI:** 10.3390/antib7030031

**Published:** 2018-08-24

**Authors:** Nina Möker, Solveig Peters, Robert Rauchenberger, Nicolae Ghinea, Christian Kunz, Antibodies Editorial Office

**Affiliations:** 1 MorphoSys AG, Discovery Alliance and Technologies, 82152 Planegg, Bavaria, Germany; moekern@gmail.com (N.M.); Solveig.Peters@morphosys.com (S.P.); robert.rauchenberger@online.de (R.R.); Christian.Kunz@morphosys.com (C.K.); 2 Curie Institute, Inserm-Tumoral Angiogenesis Unit, Translational Research Department, Curie Hospital, 75005-Paris, France; nicolae.ghinea@curie.fr; 3 MDPI, St. Alban-Anlage 66, 4052 Basel, Switzerland

The authors wish to make the following correction to the Conflict of Interests section in their published paper [1]:

“Christian Kunz and Solveig Peters are employees and Nina Möker and Robert Rauchenberger are former employees of MorphoSys AG, which owns the Ylanthia antibodies described in the publication.”

The manuscript will be updated and the original will remain online on the article webpage.

## References

[1] Moeker N., Peters S., Rauchenberger R., Ghinea N., Kunz C. (2017). Antibody Selection for Cancer Target Validation of FSH-Receptor in Immunohistochemical Settings. Antibodies.

