# Supplementary material for: Antibody Selection for Cancer Target Validation of FSH-Receptor in Immunohistochemical Settings
Source: Antibodies (Basel). 2017 Oct 18;6(4):15. doi: 10.3390/antib6040015 (PMC6698838; doi:10.3390/antib6040015)
Supplement: Supplementary file 1 [file antibodies-06-00015-s001.pdf]

A

	Blank	pos. control IgG2a	aFSHR323		MOR03207 IgG2a	
		100 nM	100 nM	10 nM	100 nM	10 nM
Blank	88	15387	1	1	132	122
hu_Serum Protein 1	88	15231	2	2	171	131
Protein A	5058	445721	5	5	174398	154927
Serum albumin (h)	91	17201	1	1	169	145
Fibrinogen (b)	79	16142	2	1	141	123
Hemoglobin (h)	78	26576	1	1	121	113
Transferrin (b)	65	15444	2	1	116	97
Antitrypsin	88	13592	2	1	110	93
Cell surface rec. 1_Lys (h)	42	9362	0	0	13657	7633
Cell surface rec. 2_Fc (h)	80	14092	89	17	88	71
Cell surface rec. 3_Fc (h)	83	13457	35	10	131	70
GFP	34	15715	2	1	56	45
Cell surface rec. 4_Fc (h)	60	16257	18	4	103	61
Fc (h)	43	18436	9	4	76	47
HKB11 vesicle	39	18707	3	2	74	46
FSHR_recomb.expressed in E.coli	74	26755	1514	2026	115	80
Pepsinogen	111	19979	1	1	181	93
Amyloglycosidase	42	14945	1	0	181	241
Trypsin inhibitor	35	15120	1	2	168	185
Cytochrome C	45	15264	1	2	117	122
Myoglobin	42	21005	1	1	226	106
Lectin	43	17301	0	0	992	160
Ovalbumin	48	18649	2	2	66	72
Trypsinogen	54	18654	2	0	53	135
Milk powder	56	16480	2	1	70	84
RNase B	57	16416	2	1	68	55
RNase A	69	27672	1	1	151	113
Lysozyme	67	15411	0	0	1085	630
anti-human Fab (Bethyl)	106	29668	15	1	624	4454
anti-human Fc	189	20972	2	3	565	106
Blank	63	18411	1	1	91	72
Blank	73	20345	2	1	51	48
			181	58		
			239			

B

	Blank	pos. control	Y010913_h/m_IgG2a		Y010916_h/m_IgG2a		MOR03207 h/m IgG2a_l	
		100 nM	100 nM	10 nM	100 nM	10 nM	100 nM	10 nM
Blank	66	14953	1	1	1	1	92	65
huFSHR - N-terminal biotinylated aa285-303	66	8787	3	2	2	2	108	73
Protein A	7333	114851	2	2	2	2	139691	133268
Serum albumin (h)	76	9171	1	1	1	2	108	78
Fibrinogen (b)	70	14201	1	1	1	1	108	63
Hemoglobin (h)	57	9292	1	1	1	2	64	46
Transferrin (b)	49	13641	1	1	1	1	96	61
Antitrypsin	88	8765	0	1	1	2	72	37
Cell surface rec. 1_Lys (h)	60	12122	0	0	0	0	101563	93240
Cell surface rec. 2_Fc (h)	98	8225	1	1	1	1	224	136
Cytokine (1)	42	15707	0	1	0	0	62	45
GFP	44	10988	0	0	0	0	91	48
Cell surface rec. 3_Lys (h)	44	12220	0	0	0	0	126761	130065
Fc (h)	134	18427	1	1	1	1	309	131
HKB11 vesicle	44	26542	0	0	0	0	898	126
huFSHR - N-terminal biotinylated aa303-322	31	10884	0	1	0	1	55	24
Pepsinogen	127	34963	0	1	0	0	264	86
Amyloglycosidase	42	14577	0	1	0	1	106	50
Trypsin inhibitor	47	16945	0	1	1	1	114	56
Cytochrome C	35	11622	0	1	0	1	113	44
Myoglobin	47	16824	0	1	1	1	79	34
Lectin	46	12877	4	2	6	3	127	51
Ovalbumin	41	19951	0	0	0	0	192	94
Trypsinogen	34	14752	0	1	0	1	113	39
Milk powder	47	16856	0	1	0	1	124	71
RNase B	55	14685	0	1	0	1	145	78
RNase A	72	15665	0	1	0	1	366	92
Lysozyme	65	12002	0	0	0	0	40198	34835
anti-human Fab (Bethyl)	112	36152	10	27	9	25	2877	1012
anti-human Fc	152	22851	1	1	0	0	2941	415
huFSHR - C-terminal biotinylated aa285-303	68	17752	814	1210	814	1268	167	77
Blank	62	16400	1	1	1	1	143	67
			15	20	18	24		
			35		42			

Supplementary Figure 1. Analysis of overall specificity of IgG molecules using Protein Panel Profiling (3P). Figure A shows the binding of the FSHR323 antibody towards a panel of 29 different proteins. Figure B shows the binding of Y010913 and Y010916 to a similar set of 30 different proteins. As assay control a "sticky" antibody was used (pos. control) and as reference, a well-characterized anti-lysozyme antibody (MOR03207) in the respective IgG format. For blank, anti-lysozyme and positive control the raw ECL signals are displayed. The values of the tested antibodies (FSHR323, Y010913, Y010916) are displayed as x-fold binding (rounded numbers) of analyzed antibody in respect to binding of the reference antibody at the same concentration. h, human; b, bovine.
